# Understanding community resilience during the drinking water contamination event on Oahu, Hawaii, 2021–2022: a mixed mode approach

**DOI:** 10.1186/s12889-024-20394-z

**Published:** 2024-11-14

**Authors:** Vidisha Parasram, Amanda R. Smith, Michele L. F. Bolduc, Jamie Rayman, Alex Poniatowski, Nicole Mintz, Madeline Jarvis, Alyssa N. Troeschel, Shanna Miko, Krishna Surasi, Charles Edge, Benjamin Gerhardstein, Diana Felton, Maureen F. Orr

**Affiliations:** 1 U.S. Agency for Toxic Substances and Disease Registry, Centers for Disease Control and Prevention Epidemic Intelligence Service, 1600 Clifton Rd, Atlanta, GA 30329 USA; 2 Centers for Disease Control and Prevention/ Agency for Toxic Substances and Disease Registry, 1600 Clifton Rd, Atlanta, GA 30329 USA; 3 Hawaii State Department of Health, 2385 Waimano Home Rd, Pearl City, HI 96782 USA; 4 Council of State and Territorial Epidemiologists, 2385 Waimano Home Rd, Pearl City, HI 96782 USA; 5 Health Resources and Services Administration, 5600 Fishers Ln, Rockville, MD 20852 USA

**Keywords:** Drinking water, Contamination, Community, Health, Resilience, Stress, Trust, Communication, Support, Needs, Emergency response

## Abstract

**Background:**

A petroleum leak into the Joint Base Pearl Harbor-Hickam water system on Oahu, Hawaii in November 2021 contaminated the drinking water of approximately 93,000 users, causing many to relocate for months. Perceptions of health and wellbeing were captured using the Centers for Disease Control/Agency for Toxic Substances and Disease Registry (CDC/ATSDR) Assessment of Chemical Exposures (ACE) cross-sectional survey in collaboration with the Hawaii Department of Health (HDOH).

**Methods:**

Responses from the ACE online survey of community members, businesses, schools, health care and veterinary care organizations during the contamination event, containing quantitative questions and qualitative information from an open text field, were analyzed. Separately, a qualitative key informant questionnaire was administered to community establishments. Thematic content analysis was used to analyze and identify prominent themes from the ACE open text field and the key informant responses that were triangulated by the quantitative data when the themes aligned.

**Results:**

Six major themes of disruption, communication, trust, stress, support, and ongoing needs were identified. Burdensome logistics from obtaining alternate water, negative financial impacts from relocation or losing business, distrust of information, perceived lack of support from response entities and uncertainty of long-term health impact caused significant disruption, stress and mental health. Individuals reported needing water, shelter, and mental health care while establishments wanted financial reimbursement and a resolution.

**Conclusions:**

The findings show that environmental disasters have significant disruptive and mental health impacts from stress. Identified themes can inform and improve emergency response and communication strategies and increase trust with community members during and after large chemical exposure events.

## Introduction

Chemical contamination of drinking water impacts community residents, businesses, and organizations reliant on the water supply. Recent examples of communities affected by chemical contamination of lead or per- and polyfluoroalkyl substances in drinking water show increases in stress, health concerns, uncertainty, distrust, and financial burdens [1, 2]. The community resilience literature offers a useful framework to examine the impact of contamination events [3, 4] and potential pathways to build community capacity to respond to future disasters. This research coalesces around enhancing both community and institutional structures [5] and their economic, social, information and communication capacity as well as a community’s competence to adapt and pivot to stress [6]. We investigated the impact of a recent contamination event impacting both civilian and military households on the island of Oahu, Hawaii, and its relation to community capacity using a mixed-mode approach.

## Background

### Water contamination event on Oahu

On November 28, 2021, residents and workers on Joint Base Pearl Harbor-Hickam, Oahu, Hawaii reported a petroleum-like smell and taste in their drinking water to the Hawaii Department of Health (HDOH) and the local poison control center. The HDOH issued a drinking water advisory for the entire base’s drinking water distribution system on November 29, 2021. It was later determined that a leak of petroleum (jet propellant-5 (JP-5)) had occurred on November 20, 2021, at the Red Hill Bulk Fuel underground storage tank facility that contaminated one of the wells. The well serviced the drinking water distribution system of an estimated 9,694 civilian and military households and establishments such as businesses, medical facilities, veterinary clinics, and schools. The contaminated well was taken offline, and an Interagency Drinking Water System team oversaw the flushing and returned the water distribution system into service. This flushing was undertaken by zones, with the drinking water advisory being amended on April 18, 2022 [7]. Many households were relocated to hotels until their homes were flushed. This time of contamination from November 20, 2021‒April 18, 2022, in the area serviced by the contaminated water will be referred to as “the event.”

The HDOH requested assistance from the Centers for Disease Control and Prevention (CDC)/Agency for Toxic Substances and Disease Registry (ATSDR) during this event to ascertain the health impact on the affected community. The ATSDR team conducted its Assessment of Chemical Exposures (ACE) investigation mid-event [8]. This analysis uses data collected during this time.

### Community resilience

**Fig. 1:**
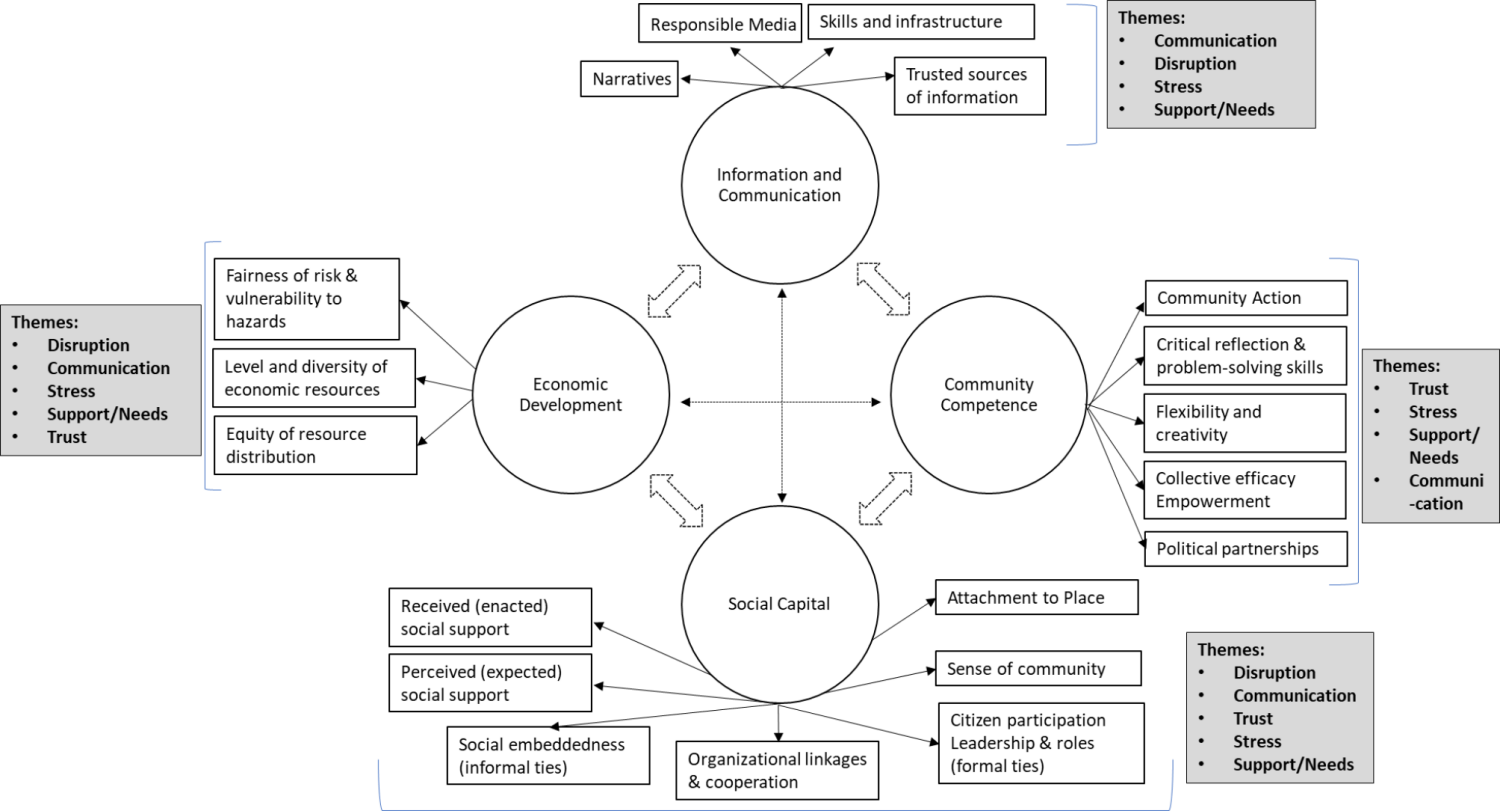
Norris et al., 2008 [9] framework of community resilience as a set of networked adaptive capacities illustrates the major themes found in our analyses of the impact of the contamination of petroleum in drinking water from community members and organizations

Community resilience is a community’s ability to respond to and recover from stressors, including environmental disasters [3, 6, 9]. The basic components of resilience are exposure, adaptive capacity, social capital, and vulnerability [9, 10]. A community’s resilience during disasters is linked to a set of networked adaptive capacities that include economic development, information and communication, community competence, and social capital [9]. During disruptive events such as environmental disasters, social vulnerabilities become more evident as communities experience stress due to the disruption of livelihood and loss of security [10]. Therefore, community resilience is a measure of how the economic, institutional, social, and ecological components of a community withstand and recover from external stress. Hence, the resilience of institutions and how they deal with external pressures and stress during an emergency may determine how a community recovers from such events [11]. This paper documents the various adaptive capacities present in themes identified through the ACE surveys to inform planning to ensure communities are adequately prepared and more resilient when faced with similar future incidents, including natural disasters. Figure 1 uses Norris et al., 2008’s community resilience framework to assess the adaptive capacity of the community affected by the 2021 petroleum leak.

## Methods

### Study population and design

Three sources of data were used for these analyses. The CDC/ATSDR ACE response team created an online survey adapted from the ACE toolkit interviewer-administered general survey in Epi Info. This survey was open from January 7 through February 10, 2022. Community members potentially affected by the contaminated water from the initial date of the event through the date of survey closure, including both military and civilian households, workers, and school children, were invited to complete an ACE online health survey. A flyer with a QR code containing the survey link was shared with residents at water distribution sites, door-to-door on Joint Base Hickam Pearl Harbor, at local schools, and via the Oahu Department of Health website. Parents and guardians completed the survey on behalf of persons aged < 18 years. Relevant questions from the ACE online quantitative (AOQ) portion of the survey are presented in Table 1 and are the first source of data. The ACE online text (AOT) final question, “Is there anything that we did not cover that you want to tell us related to the incident?” was used as the second source of data. Responses concerning health effects and pets’ health are reported in Miko et al., 2023 [12]. This activity was reviewed by the CDC and was conducted consistent with applicable federal law and CDC policy.

**Table 1:** Questions in the ACE online survey quantitative analyses by theme and sub-theme

Theme	AOQ questions analyzed
Disruption	Confidence in safety of tap water — How confident are you that your tap water will be safe to drink in the next 30 days? (10-point scale)
	Missed School — Did your child miss school because of the incident (e.g., school closure, transportation issues, kept child home)?
	Missed Work — Was your business/employment negatively impacted by the incident?
	Concern about going to work — Have you have any concerns about going to work because of the incident?
Communication	How did you first learn that the incident could affect you and your family?
Trust	Concern about safety of tap water
	Before incident — How concerned were you about the safety of your tap water BEFORE learning of the incident? (10-point scale)
	After incident — What is your level of concern about the quality and safety of your tap water AFTER learning of the incident? (10-point scale)
	Confidence in safety of tap water — How confident are you that your tap water will be safe to drink in the next 30 days? (10-point scale)
Stress	No Corresponding Question in ACE Survey
Support	Support from Neighbors — How much support have you received from neighbors and other community members or groups since the incident? (10-point scale)
	Support from Gov — How much support have you received from government entities since the incident? (10-point scale)
Ongoing Needs	As a result of this incident, are you or your family in need of any of the following: (Check all that apply: water, medical care, shelter, mental health care, other).

A key informant questionnaire, (KIQ) (Table 2), was administered using a purposeful sampling strategy. ACE team members contacted various sectors in the community through email, phone calls, and in-person outreach to encourage them to participate in a 10–15-minute questionnaire. Surveys were self-administered, and whenever possible, team members used electronic data capture. Participants were employees or representatives of various sectors. Individual participants of the AOT were also able to represent a sector in the KIQ. Participants were deidentified in both the AOT and KIQ prior to analysis. We reached out to 73 informants, of whom 39 (52.7% response rate) responded: businesses (*n* = 14), schools/daycares (*n* = 10), medical facilities (*n* = 6), community groups (*n* = 3), veterinary clinics (*n* = 1), and others (*n* = 5). Restaurant managers completed the most questionnaires among businesses, followed by elementary school administrators, staff members from healthcare facilities, and a dental office.

**Table 2:** Relevant KIQ questions by group

Group	KIQ Questions
All Sectors	How is your organization or business impacted by the Navy water contamination? Please describe.
	Did you experience challenges to keeping your business or organization open during the water contamination incident?
	Describe some of the challenges your organization or business faced during the water contamination incident.
Businesses	Has your organization or business laid off workers as a result of the water contamination incident?
	If your organization or business laid off workers, please describe why your business or organization needed to do so.
	How many workers did your organization or business lay off?
	Has your organization or business had to scale back work schedules?
	Has your organization or business implemented new procedures?
	What is the financial impact of the spill on your organization or business?
Schools and Childcare	Did your school or daycare have students call out sick?
	How is your school or daycare providing water to students and staff?
	Has the school nurse observed an increase in illnesses related to the water contamination incident?
Health Care	Did your practice see an increase in symptoms among patients who visited your office?
	Did you see patients with concerns about the contamination? If so, please explain.
	What are the symptoms being observed?
	What advice are you sharing with your patients?
All community Groups	What do community groups need to support community members?
	Based on your experiences and observations in your organizational role, how has the water contamination been handled so far? Please explain what has not worked well.
	Based on your experiences and observations in your organizational role, what activities or organizations have been helpful in responding to the water contamination?
	Based on your experiences and observations in your organizational role, do community members trust the process and organizations involved in responding to the Navy Water System contamination, so far? Please explain.
	Describe any stress you are experiencing in your organizational role from this water contamination incident.
	How is your organization or school or business being supported? Please describe.
	Who is providing your organization or school or business with support?
	What are the current needs of your organization or school or business to address the water contamination? Please describe.

### Qualitative analysis

Thematic content analyses were used to assess the qualitative data from the AOT and the KIQ. The responses were first examined and coded by a team of researchers who then identified preliminary emerging themes from the data. Overarching themes and subthemes were defined in an Excel codebook and used by all coders: long-term health, disruption, communication, trust, stress, ongoing needs, financial burden, and pets’ health, among others. Responses were then coded by a team of 4–6 coders. Other emerging themes and codes and subcodes were documented and folded into the iterative codebook. Any new emerging theme or discrepancy in codes was discussed, and agreement was reached before use. Intercoder reliability was achieved by double coding using pairs of coders who examined both sources. Major thematic findings from the responses are paraphrased and presented using representative quotes in Table 3. Quotes are presented verbatim with minor edits to improve clarity. The themes were then analyzed using Norris et al., 2008 community resilience framework to demonstrate the adaptive capacity of the affected community.

Finally, quantitative analyses of the AOQ pertaining to the identified themes were performed using R (Version 4.2.0). Answers were not required for all questions on the ACE survey, so the total number of respondents varied by question. The initial quantitative findings from the ACE survey of this community were previously reported [8].

**Table 3:** Representative quotes from the qualitative analysis for each of the themes and sub-themes

Theme	AOT Representative Quotes	KIQ Representative Quotes
Disruption	“Missed a lot of work due to being sick from the water. Even ended up catching covid because i am now living in a hotel surrounded by tourists and many other sick people when I should be living in the house that my wife works and pays for. Our BAH is not being refunded for the two months we have not been living in our home. Little has been done to make sure families are safe from the pandemic that is still very much going on on top of the continual lies and misinformation that is being put out by the military.” “ I have picked up water from the water distribution center a couple of times and it is very convenient. I am not crazy about the plastic bottles but I am very happy about the refillable jugs.”	“Drastically, had to change system for everything, ice beverages dishwashing hand washing. It’s been very detrimental.” “We have been paying all our workers for the first 2 months. We are now critical on cash as we cannot get help from the Navy, […], nor our insurer. We had to stop paying some staff because we are out of cash. We are concerned we will be unable to reopen when [facility management…] gives us the ok to reopen, because we will not have staff.” “The water contamination has disrupted our school since 11/29/21. We have no running water. Water has to be brought into our school. Access to the water needs to be available to students and staff (500 people). Our lunch service has also been disrupted by this as well. We cannot use water to cook with as well. Students have not been able to get to school on time because they are in temporary lodging […].”
Communication	“We had to find out via social media, which was not until we were already two weeks into the discovery of the issue.”	“A lot of requests for information/questions from patients + parents of children of what could possibly happen to them long term due to the contamination. But unable to provide that guidance, as no helpful answers have been provided from experts or DoD.”
Trust	“Since May of 2021 I have noticed an oil sheen in my water when filling pots to cook with.[…] Please investigate when other spills may have happened. I do not believe this was an isolated incident and I no longer trust a word from the Navy.” “The initial response from the Navy leadership led us to believe that the water was safe. Because of that, we continued to drink the water for several days, until skin issues and headaches appeared. I am extremely disappointed in the JB leadership and no longer trust that military leadership are primarily concerned with my family’s health and well-being.”	“We have been doing our best but our personnel has been stretched thin. The military has made lots of promises but many of them were empty promises.” “A major loss of trust has been observed, but this has particularly been in the Navy. On a larger scale, a loss of trust in the military as a whole. Significantly reduced amount of trust due to a lack of information provided early on in the incident, and a lack of accuracy as to what was provided. So now, consequently, people are having a difficult time trusting what officials are saying today.”
Stress	“I live in an ADA unit […]. Packing up and moving to a hotel was out of the question because of this, also where would my dog go? I’m in no financial position to front the cost of a hotel, pay for food, boarding for my dog, rent + utilities. It’s incredibly disheartening and downright wrong how concerns were not being heard enough to investigate the situation. We’ve requested our water at our home to be tested and no one has contacted us. Every home in these communities should be tested! This has caused so much undue stress!”	“We are facing bankruptcy. Our stress level is through the roof.”
Support	“The lack of support for those who aren’t active military is absolutely horrendous. The navy poisoned us & the least they could do is help out with expenditures & taking accountability.” “Is there a timeline? It’s getting increasingly frustrating not knowing when this is over and we can safely return home. Paying high rent each month for a home we can’t live in is just absurd! Thank you for doing this survey and giving us a voice!” “Seeing as that we were unable to live in our house that we pay rent for, I would like to see some sort of reimbursement for this. Negligence of this magnitude should never fall on those affected. I understand lodging was procured on our behalf, but that does not justify taking all of my entitled BAH to store my household goods in a 1600 sq ft storage unit.”	“We are relying on the restaurant relief fund we received from SBA to support the business.” “Clean running water!!!! Manpower to transport water and refill hand washing stations more frequently and consistently.” “We need this problem solved, we need a job fair to hire new employees, we need a break/assistance from the government side or Navy side to give us rent assistance to get back on our feet. When they shut us down, it was so sudden that we had to throw away thousands of dollars’ worth of inventory. Inventory and money we will not be able to recoup due to the water contamination.” “As physicians, we need guidance on what health impacts to monitor for long-term, and how to monitor for long-term health impacts of this exposure for patients.”

## Results

**Fig. 2:**
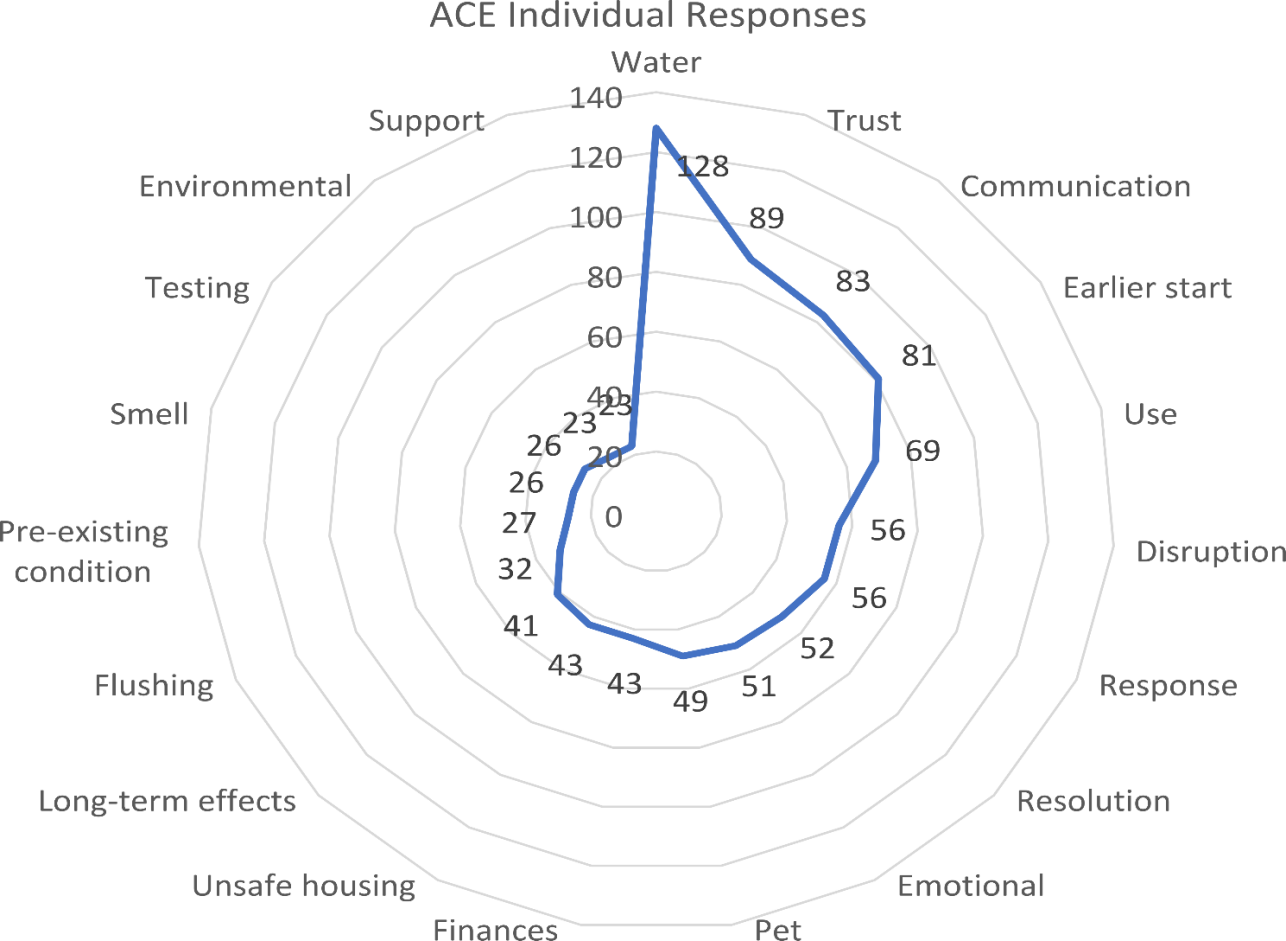
Responses aligning with major themes and subcodes from ACE survey open text responses

A total of 2,289 eligible participants submitted AOQ surveys from 1,389 (14%) of the 9,694 estimated affected households [8] with 733 open text responses. The analysis identified six common themes: disruption, communication, trust, stress, support, and ongoing needs. Themes were discerned from the AOQ questions listed in Table 1 and the qualitative responses from the KIQ in Table 2. Additional themes also emerged that informed our major themes but were not represented in either source. These are described under the “Other” theme. Some themes are not presented in this paper because they were only available in the ACE online survey open text field. These included prominent themes surrounding health symptoms and water and minor themes such as inequity that emerged from the qualitative data and are especially important when describing the symptoms, experiences of drinking water loss, or differential treatment of military versus civilian community members. Figure 2 shows the frequency of subcodes and how they corroborate with themes from the AOQ open text fields.

### Disruption

AOT results showed that people reported reoccurring symptoms after returning to the affected area and needing to alter their lives due to illness. They raised concerns about feeling stuck in their rental lease agreement despite having to relocate due to the contamination event. Community members also reported disruption due to water distribution system flushing activities occurring in their homes. While some participants reported that their symptoms improved after they changed to another water source or their homes were flushed, others reported additional or worsening health symptoms after flushing. Table 4 shows the major themes and subcodes obtained from the ACE open text fields. Many survey respondents described noticing odors after the flushing, which exacerbated symptoms.

The KIQ results showed that community businesses and organizations also experienced disruptive effects on their daily operations. Both business and school respondents reported being overworked and overburdened from developing and implementing new procedures and obtaining water from an alternative source to maintain operations. Responses indicate that the event resulted in significant financial impact. Restaurant respondents reported significant financial loss from closures, losing customers due to missing the peak shopping months, buying water, and losing food, contributing to their financial burden. Some businesses reported having to cut staff hours or lay them off or struggling to continue to pay them. A few business respondents reported experiencing a boost in business during the contamination but with the added pressure to keep up with the increased businesses without an onsite water supply. Some schools also had to reapportion the responsibility of staff to address water distribution needs. One school representative explained:

“We have no running water. Water has to be brought in […] lunch service has also been disrupted […] cannot use water to cook […]. Students have not been able to get to school on time […] and sometimes have not eaten breakfast. We allow for them to eat which decreases their seat time in class […] volunteers every day for water maintenance […] [means] there’s less parking […].”

Health care providers reported disruptions to their organizations’ regular water use patterns and needing to develop informal procedures to meet their water needs while caring for patients. Respondents from other organizations (a religious organization, a fire station, a food service entity, and a military organization) also reported implementing new procedures and experiencing displacement and disruption.

AOQ supported the disruption theme. Many respondents had to change their patterns of water use, including drinking, cooking, hygiene practices, and pet care. Almost half (*n* = 504, 48%) of 1,060 adult respondents from the ACE survey reported having their business or employment negatively impacted by the incident; a third (*n* = 362, 33%) reported concerns about going to work because of the incident. Among children aged 6–17 years, 41% (*n* = 161 of 396 with valid responses) missed school because of the incident.

**Table 4:** Primary constructs addressing resilience from the ACE open text responses among community members and their associated themes

ACE Text Primary Themes	Associated Codes
Disruption	Symptoms
	Water
	Emotional response
	Use
	Finances
	Loss
	Communication
	Testing
	Trust
	Long-term effects
	Resolution
	Unsafe housing
	Stress
	Pre-existing conditions
Communication	Trust
	Water
	Symptoms
	Finances
	Use
	Emotional Response
	Disruption
	Earlier start
	Location-specific
	Loss
	Long-term effects
	Flushing
Trust	Communication
	Symptoms
	Water
	Response
	Emotional
	Flushing
	Environmental
	Use
	Earlier start
	Testing
	Unsafe housing
	Disruption
Stress/Emotional Needs	Symptoms
	Disruption
	Stress
	Communication
	Trust
	Unsafe housing
	Long-term
	Effects
	Pre-existing condition
	Finances
	Anxiety
	Water use
	Resolution
	Earlier start
Support/ Ongoing Needs	Finances
	Loss
	Disruption
	Communication
	Symptoms
	Water
	Inequity
	Trust
	Unsafe housing
	Lack
	Environmental
	Response
	Testing
	Use

### Communication

Another major theme identified was communication gaps, including issues around initial notification about the incident, flushing of the water distribution system, and updates following the incident.

AOT noted that some reached out to the Department of Health to report health issues but did not receive responses. Many respondents expressed concern that the pace and tone of communications from the responsible party mismatched the alarm, disruption, and changing conditions of the situation. They expressed that they wanted immediate information from the responsible party and reported relying on their neighbors instead to find clean water. At the time of the online survey, flushing and water testing had occurred at some of the residences in the affected area, but the households had not yet been informed of the results. Residents also wanted more information on the selection of locations to conduct flushing and the results. They did not understand why a neighboring street of homes was flushed, while other nearby residents were told it was safe to return to their unflushed homes. Furthermore, participants stated the lack of information on how to address immunocompromised people left them guessing how to take care of sick family members. Table 3 contains quotes aligning with the major themes.

KIQ expressed a lack of information and instructions from official government sources about water distribution system flushing. Restaurant respondents wanted to know when to use the water after flushing, while healthcare providers wanted to obtain water testing results to understand the exposures faced by their patients. Some community members deemed the sharing of information and communication about flushing inadequate. Many identified getting initial information on flushing from social media rather than from official sources. Others found that the responsible party did not clearly articulate flushing directions to the impacted communities:

“Poor direct communication from the navy. Facebook should not be the desired mode of communication….”

Healthcare providers felt they did not have the appropriate information to answer patients’ questions and lacked sufficient information on what health impacts to monitor. They wanted additional guidance and information to provide to patients about the incident and its potential long-term effects:

“As physicians, we need guidance on what health impacts to monitor for long-term, and how to monitor for long-term health impacts of this exposure for patients.”

Community groups also reported gaps in communication to the residents they served and distrust in the information communicated to the community from response authorities. One organization discussed confusing communication during the initial days of the spill.

“…There was a breakdown in communication on day one of the first hosted community meeting […]. The guidance from the US Navy differed for many weeks before they finally adhered to [Department] of Health guidance, prior to this they would make comments that “their experts” in each of the armed forces branches provided such guidance which was not the same as [the Department of Health]….”

AOQ revealed reliance on nonofficial sources of information. Approximately half 908/1,791 (51%) of respondents indicated first hearing about the incident via social media; 19% (347) of respondents indicated first hearing about it from a relative, friend, neighbor, or coworker; 118 (7%) reported first learning about the incident directly from an authority; and 27% (490/1,817) reported obtaining their information from a website. Over a third, 38% (*n* = 684) of respondents reported feeling that they had the information needed to feel comfortable making choices regarding the incident.

### Trust

Responses from all sources indicated a loss of trust in information, authorities, and actions related to the incident. This included a lack of trust in the information being shared about the incident, a lack of trust in the safety of the tap water, and a mistrust of the declared start date of the incident being November 2021.

AOT also linked their erosion of trust to a lack of transparency and concerns about the quality and clarity of information:

“The initial response from the Navy leadership led us to believe that the water was safe. Because of that, we continued to drink the water for several days until skin issues and headaches appeared. I am extremely disappointed in the JB [Joint Base] leadership and no longer trust that military leadership is primarily concerned with my family’s health and well-being.”

Individual respondents felt that water contamination began earlier in 2021 or even in prior years.

“Since May 2021, I have noticed an oil sheen in my water when filling pots to cook with. […]Please investigate when other spills may have happened. I do not believe this was an isolated incident, and I no longer trust a word from the Navy.”

Individuals reported distrust of some housing providers, whom residents felt were being disingenuous in their communications about the contamination of their water and later flushing activities. Some respondents cautiously returned to using the water after flushing. They stated that while they started to shower and do laundry with the water, they still did not trust it for cooking or to bathe their children.

Several respondents expressed significant concern with the contamination of their PVC piping. They relayed distrust in the safety of the water and expressed concerns about long-term health effects due to ongoing contamination.

“Concerned about long term effects of issue or future problems we will encounter. We definitely will not trust the water from here on out and plan to leave the island once our lease is up. […] The pipes need to be replaced, and simply flushing them will not completely eradicate the problem. The contamination of an oil-based/petroleum solution in any oil-based pipe such as PVC or mixing valves with oil-based products such as rubber or plastic will always be contaminated. The only safe solution is to replace everything.”

Some individuals stated that they wanted their home’s water tested after the flushing, and some reported smelling an odor from their water after the flushing. Others were concerned that the Navy was not testing for all the chemicals that were released. Some indicated that sharing sampling results would assuage their concerns.

Several KIQ responses also expressed a lack of trust and transparency in the communication and the entities responsible for the response, for example, from official military sources:

“I feel that they are not telling us the truth and extent of the damages, they are stringing us along. Therefore, we cannot convey the correct information to our employees.”

Some, but not all, health providers expressed distrust, especially of the military information being shared.

The AOQ asked the respondent to rate the level of concern for the quality and safety of their tap water on a scale of 1 (lowest concern) to 10 (highest concern). Individuals participating in the quantitative ACE survey reported a low level of concern (median: 2; range: 1–6) about the quality and safety of their tap water *prior* to learning of the incident and reported the highest level of concern (median: 10; range 10–10) about the quality and safety of their tap water *after* learning of the incident. Additionally, respondents reported a very low level of confidence (median: 1; range: 1–2) that their tap water would be safe to drink in the next 30 days.

### Stress

Other identified themes led to increased levels of stress for community members and organizations. Individuals had to grapple with personal, professional, and financial impacts.

AOT noted a military respondent who spoke of stress from fear of losing their job for speaking out against the military response.

Respondents indicated stress through concerns about the potential long-term health and environmental impacts. Some wanted the event documented in their permanent medical records in case related health issues arise later. Another respondent was concerned that health care providers were not conducting specific tests to understand whether their symptoms were due to exposure to the contaminated water. Respondents stated that without documenting illnesses caused by contamination, military families could not apply for reassignment.

Stress was further exacerbated for some who expressed environmental concerns about the disposal of flushing water being pumped into lagoons or the ocean. Some worried about smelling fuel from the disposal of flushing water on their lawn and exposing children and animals. One reported making dozens of complaints about soil contamination from the discharge of contaminated water from flushing and sprinklers. Others expressed frustration with the poor administration of the flushing plan, such as spilling flushing water in their homes, skipping steps, water leaks after pipes were flushed leading to mold, and flushing personnel entering their home.

Others expressed worry about the occurrence of a similar event in the future if the Red Hill storage tank facility did not close.

The KIQ results showed stress from financial loss, extra work, increased business due to the closure of surrounding establishments, needing to find water to keep their businesses running, educating patrons on water use, and changing operating procedures. Additionally, among restaurants and food service establishments, management of the response and lack of communication and information related to the response emerged as consistent themes related to stress. Many respondents also identified not having a date of resolution as a major source of stress.

School KIQ data indicated high levels of stress associated with the changing procedures described above, compounded by dealing with the contamination incident during the COVID-19 pandemic.

### Support

Support was another theme expressed in the analyses. Both community members and organization respondents reported feeling under supported during the water contamination event.

AOT noted that some people felt a lack of support for civilians and nonactive military service members compared with active military personnel.

A military AOT respondent reported an excess financial burden despite receiving military assistance because of waiting for reimbursement of upfront costs, which was not guaranteed in full. Others reported delays in the distribution of water and information to their community.

Many responses reflected multiple overlapping themes. Some participants expressed sentiments falling into support and communication. Responses from many individuals indicated that they felt they received very little support from government entities or the housing development where they resided during the incident. Some initially received water from their housing development, but support waned, and people ran out of bottles. Many community members were grateful for the services provided by response entities despite identifying logistical and communication challenges.

“Thank you for providing water stations and laundry service, though the whole issue is a great inconvenience, this was necessary to have available. The communication piece was a failure during this incident. The website and phone numbers I found on your main updates page did not have clear lines of communication. […] That would be my biggest advice; talk and let people know what’s going on through the Navy first before we hear it from our neighbors. Thank you.”

KIQ responses identified a lack and variation in support, including government (water logistics, flushing) or financial support. Most notably, they felt that poor communication augmented the loss of financial support. They also relayed frustration with the delay in response when they needed assistance.

KIQs from schools were more likely to express having received support than those from businesses. School officials reported receiving support from the military, with some schools reporting assistance from local nongovernmental and faith organizations. Other schools found their complex area superintendents — a school superintendent with jurisdiction over schools located on military bases — and military liaisons essential to ensuring they received water and other resources. School principals also reported receiving support from volunteers from the military who delivered water to the schools.

KIQS from organizations other than schools generally felt less supported because they did not have an existing local network. Some business informants reported obtaining financial support from the Small Business Administration (SBA), corporate offices, facilities or property management or other larger entities, while others reported receiving no financial support.

During flushing activities, restaurant informants expressed not receiving any support from government entities to conduct flushing and having to rely on the plumbers provided by their property center for flushing.

When asked in AOQ to rank their level of support from 1 (lowest support) to 10 (highest support), respondents reported feeling moderately supported by their community (median: 6, range: 3–8) and government entities (median: 6, range 3–8).

### Ongoing needs

In the AOT in some communities, individual respondents needed access to drinking water and information on contamination but reported a continued lack of support from response entities.

KIQ respondents from restaurants and other businesses whose livelihood depended on water reported numerous ongoing needs, including wanting a resolution to water contamination, reimbursement, better communication, issues with employment, and financial assistance. The uncertainty of not knowing when the water would be available curtailed any planning. For example, one recycling facility that utilizes water in its cleaning process needs water quality and availability assurances to continue operations.

To meet the needs posed by financial losses, respondents from other businesses asked for compensation for their losses or an extension of their contracts:

“We need this problem solved, we need a job fair to hire new employees, we need a break/assistance from the government side or Navy side to give us rent assistance to get back on our feet. When they shut us down, it was so sudden that we had to throw away thousands of dollars’ worth of inventory. Inventory and money we will not be able to recoup due to the water contamination.”

School respondents reported needing additional support to ensure a consistent water supply and to distribute the bulk water provided and service sanitation stations, as well as needing reimbursement for the time and resources used to address the event. One school respondent requested transportation for the students displaced and staying in hotels.

Healthcare providers sought additional guidance on the long-term effects of the exposure to provide information to their patients and better communication and information on when the water is safe to use.

Respondents from other community and faith-based groups and nongovernmental organizations expressed needing financial assistance, flushing of their neighborhoods, water testing to ascertain community exposure, and more clean drinking water to alleviate the impact of the spill. A need for information about the type of medical tests and labs to identify exposure was reported from a military organization. Many expressed a need for resolution and financial support.

Water was the most identified ongoing need during the contamination event by AOQ (*n* = 850, 47%), followed by shelter (*n* = 190, 10%), mental health care (*n* = 186, 10%), and food (*n* = 164, 9%). Approximately one-third of the respondents reported not needing anything.

### Other themes

Our study identified additional thematic subcodes that were not prominent or represented across all three data sources.

*Inequity* AOT noted inequities related to differences in assistance between civilian and military residents with bottled drinking water, housing relocation, and general assistance. One individual commented that very little information was being provided about risk to individuals who work in areas affected by the spill but live off base. Some noted inequities in level of support across different military branches, different information about which geographical communities were affected by the spill and what actions to take if affected, different dates on-base preventive actions were taken, and differences in when temporary lodging allowance and per diem were being offered to affected service members. A KIQ respondent commented on differences in how military and civilian families were treated, and a school official reported that inequitable treatment of families led to difficulties at school.

*Unique life situations* A few AOT respondents mentioned challenges they faced due to unique life situations, such as being unable to temporarily move out of their residences. A military retiree felt forgotten and expressed the need for more information and compensation. One resident chose to stay in the affected area despite having the option to move because it would drastically change the daily routine for their special needs child.

## Discussion

To our knowledge, our study is the first to use a resilience framework to evaluate the responses of community members and organizations during a contamination event. Although the intent of our study was not to test Norris et al., 2008 or other community resilience frameworks, this literature base guided our analysis and the framing of our findings. Norris et al., 2008 identified information and communication, community competence, social capital, and economic development as the four major constructs in the network of adaptive capacities that enable a community to be more resilient during disruptive events. Our findings contribute to understanding each capacity within a water contamination event. Our qualitative analyses revealed six common themes related to the incident: disruption; communication; trust; support, ongoing needs, and stress. Respondents reported economic and logistic burdens as well as emotional and psychological distress caused by ongoing contamination. Culberson et al., 2016 also documents an increase in stress and anxiety among residents in Flint, Michigan 12 months after the lead contaminated the city’s drinking water [13]. Additionally, a 2014 Community Assessment for Public Health Emergency Response (CASPER) questionnaire conducted after the 4-methylcyclohexaemethanol and propylene glycol phenyl spill affecting Charleston, West Virginia drinking water for 2 weeks found 3.5% (*N* = 4,175) of households reported at least one person experiencing mental health issues related to the chemical spill [14].

Improving communication can improve other themes, such as trust and stress. Reported best practices for incident management to build trust include but are not limited to coordinating communication and action across agencies, using shared communication channels, minimizing conflicting information, and ensuring equity in the response across the affected population [15, 16]. Long-term actions to build trust include clearly explaining in plain language the cause of water contamination, communicating planned steps to prevent future incidents, implementing a robust water quality monitoring plan, and offering on-demand water testing for system users. In addition, being transparent and timely with results and updates is essential in building community trust. The KIQ data showed that the need for information to facilitate operations varied by the different establishments during this event, which may inform more effective responses to future events. Further, a systematic review of the psychological impacts of chemical, biological, radiological or nuclear events finds conflicting or unclear messaging from emergency response authorities increases uncertainty and psychological problems and advises clear and consistent risk and crisis communication from governmental and public health organizations [17].

Water contamination events are known to contribute to long-lasting mistrust in drinking water supplies and changes in water consumption behaviors that have financial, health, educational, and environmental impacts [1]. Our findings are consistent with these other drinking water contamination incidents [1, 2] and may persist for some time after the event [18].

Our results indicated that most of our respondents received their information from electronic sources, including social media. Notably, this suggests that developing and initiating a social media strategy at the onset of an incident can be an effective way to directly communicate information to the public. Future emergency responses could consider developing a multimedia communication plan with a dedicated person of authority to build trust through transparency. The plan would be used throughout a response to consistently communicate both response and mitigation strategies to the public.

Our analyses showed that school respondents reported receiving more support than businesses and other smaller community organizations. The presence of an existing military liaison within the schools on-base facilitated communication and requests for resources. Developing community- and individual-focused preparedness plans that establish networks of volunteers and organizations who can assist during water contamination events can lead to better support for schools and other organizations in future emergency events. Exploring ways to provide support to other organizations during emergency events is needed.

Meeting community needs during an emergency response is essential to mitigating the disruptive effects of a catastrophic event. Most of our study participants (individuals and organizations) identified needing clean and accessible water, financial support and compensation, transparent communication about the incident and response activities, and a quick resolution to the incident. Furthermore, our analyses identified a subtheme of differential access to communication. Some community members expressed feelings of isolation and disregard during the event. Our study did not specifically seek information on differential treatment received during this event, but future studies examining other emergencies affecting similar communities could explore the depth of this differential treatment observed by study participants.

Finally, our study had several limitations. Our selection methods may have selection bias, as participants were more likely impacted and, therefore, more apt to report symptoms and effects of the contamination. Our use of different modes via the individual level and community organization questionnaire may also introduce some response bias. Additionally, the KIQ purposeful sampling strategy may not be representative of all the establishments impacted by the event.

Our data are reflective of the first two months of an ongoing event, not later changes in support, trust, or stress. In September 2022, 6 months after the contamination was remediated, a second ACE was conducted including more in-depth qualitative questions with similar results [19]. Additional investigations to understand the ongoing and/or long-term impacts of the contamination event are ongoing. We anticipate that future studies will continue to characterize community resilience during the response.

## Conclusions

Our study uses the resilience framework to assess community survey responses regarding impacts from chemical contamination of a large drinking water system in Oahu, Hawaii, for approximately 3 months. Qualitative findings from both community and key informant surveys, supported by quantitative results, demonstrate the importance of consistent, transparent, and accessible communication from officials to ensure community trust. Our data also identify the importance of having strong networks of financial and social support during the response. Finally, our findings increase the understanding of the constructs of resilience by describing the community’s experience of disruption, communication, trust, stress, support, and ongoing needs during a contamination event.

## Data Availability

Data is provided within the manuscript or supplementary information files. Those interested in accessing the data may contact the corresponding author Vidisha Parasram at vparasram@cdc.gov.

## Abbreviations

CDC/ATSDR: Centers for Disease Control/Agency for Toxic Substances and Disease
Registry ACE: Assessment of Chemical Exposures
HDOH: Hawaii Department of Health
JP-5: Jet propellant-5
AOQ: ACE online quantitative
AOT: ACE online text
KIQ: Key Informant Questionnaire
